# Entrezpy: a Python library to dynamically interact with the NCBI Entrez databases

**DOI:** 10.1093/bioinformatics/btz385

**Published:** 2019-05-11

**Authors:** Jan P Buchmann, Edward C Holmes

**Affiliations:** Marie Bashir Institute for Infectious Diseases and Biosecurity, Charles Perkins Centre, School of Life and Environmental Sciences and Sydney Medical School, The University of Sydney, Sydney, NSW 2006, Australia

## Abstract

**Summary:**

Entrezpy is a Python library that automates the querying and downloading of data from the Entrez databases at National Center for Biotechnology Information by interacting with E-Utilities. Entrezpy implements complex queries by automatically creating E-Utility parameters from the results obtained that can then be used directly in subsequent queries. Entrezpy also allows the user to cache and retrieve results locally, implements interactions with all Entrez databases as part of an analysis pipeline and adjusts parameters within an ongoing query or using prior results. Entrezpy’s modular design enables it to easily extend and adjust existing E-Utility functions.

**Availability and implementation:**

Entrezpy is implemented in Python 3 (≥3.6) and depends only on the Python Standard Library. It is available via PyPi (https://pypi.org/project/entrezpy/) and at https://gitlab.com/ncbipy/entrezpy.git. Entrezpy is licensed under the LGPLv3 and also at http://entrezpy.readthedocs.io/.

## 1 Introduction

The increasing availability of biological data has not only resulted in a multitude of genome sequence data, but also substantial increases in the amount of accompanying metadata, including phylogenies, sampling conditions and locations and gene ontologies. To use such data in a biological analysis pipeline a programmatic approach is required to query and retrieve data from these databases. The National Center for Biotechnology Information (NCBI) is one of the largest such repositories and both developed and maintains the Entrez databases that currently comprise 37 individual databases storing 2.1 billion records related to the life sciences (NCBI Resource Coordinators, 2016).

NCBI offers two approaches to interact programmatically with its Entrez databases: (i) E-utilities (http://eutils.ncbi.nlm.nih.gov/) are a set of tools that allow the user to query and retrieve NCBI data using specific Uniform Resource Identifiers (URIs). Entrez databases can be accessed using an URI describing the function and its parameter, such as searching a database with a specific term; and (ii) Entrez Direct—a powerful Perl program that allows *ad hoc* access to the NCBI databases through a command line interface (Kans, 2016, https://www.ncbi.nlm.nih.gov/books/NBK179288). E-Utilities offer a low-level interface to the Entrez databases via Entrez Direct. However, Entrez Direct is designed as a command line tool and is therefore primarily incorporated into analysis pipelines via a Shell, such as Bash, but not designed as a library. Although Python is increasingly used by biologists, incorporating Entrez Direct into Python pipelines requires the use of new processes outside Python, adding an additional layer of complexity.

Herein, we present Entrezpy. To our knowledge, this is the first Python library to offer the same functionalities as Entrez Direct, but as a Python library. Existing libraries, such as Biopython (Cock *et al.*, 2009) or ETE 3 (Huerta-Cepas *et al.*, 2016), offer either a basic or a very narrow interaction with E-utilities. Biopython does not handle whole queries, leaving the user to implement the logic to fetch large requests, while ETE represents a library focusing only on phylogenetics. In contrast, Entrezpy is specifically designed to interact with E-Utilities. It offers fine grained control on how to download data and can cache results locally for quick retrieval. This allows the querying and downloading data from Entrez databases as an integral part of an analysis pipeline. Entrezpy automatically configures itself to retrieve large datasets according to the implemented E-Utility function and limits enforced by NCBI.

Entrezpy includes a helper class, termed Conduit, that facilitates the creation and execution of query pipelines; that is, several consecutive queries that may depend on previous queries with possible dependencies, and the ability to re-use previously obtained results. Entrezpy is licensed under the GNU Lesser General Public License and is packaged in PyPi (https://pypi.org/project/entrezpy/) or can be obtained from https://gitlab.com/ncbipy/entrezpy. The Entrezpy source code is documented using Sphinx (http://www.sphinx-doc.org/en/stable/index.html) and the documentation, including usage examples, is available at https://entrezpy.readthedocs.io/.

## 2 Implementation

**Fig. 1.**
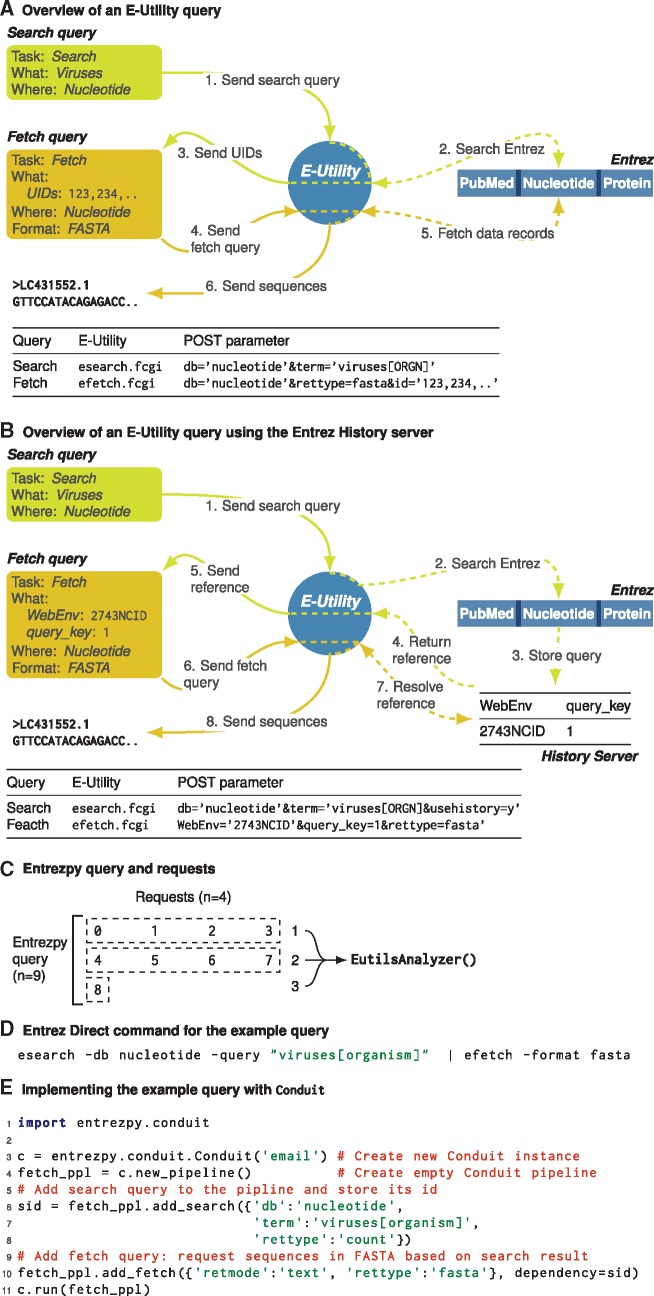
E-Utility examples using two queries to download virus sequences from the Nucleotide Entrez database. (**A**) E-utility steps without using the Entrez History server. (**B**) The E-utility steps when using the Entrez History server. Tables in A and B list the minimum required POST parameter for queries sent to E-Utilities. All E-Utility functions start with https://eutils.ncbi.nlm.nih.gov/entrez/eutils/. Dashed lines indicate the figurative and not literal interaction between E-Utilities and Entrez databases. Only three Entrez databases are depicted. Numbers indicate the sequence of steps in the query. (**C**) Depiction of Entrezpy queries and requests using a query with nine data records and a request size of 4. The size of the last request is automatically adjusted. All requests within a query are passed to the same Entrezpy EutilsAnalyzer instance. (**D**) Resolving the example queries using Entrez-Direct. (**E**) Resolving the example queries using Conduit

Data records within an Entrez database are identified by their identification number. The Entrez documentation refers to this number interchangeably as either an UID or ID. For the remainder of this article we will use the term UID to refer to a data record identification number. UIDs are unique within an Entrez database but not across Entrez databases. Entrezpy is a library of Python classes implementing the specific steps required to interact with the E-Utilities. Querying and downloading data via the E-Utility is achieved by sending queries encoded as an URI for the specific function and the corresponding parameters (Fig. 1). For example, the following URI searches the nucleotide database for all virus nucleotide sequences and returns the UIDs identified: https://eutils.ncbi.nlm.nih.gov/entrez/eutils/esearch.fcgi? db=nucleotide&term=viruses[orgn] (Fig. 1A).

The E-Utility returns a response describing the search result. This includes the number of data records found within the requested database and corresponding UIDs. To fetch the data records, a second E-Utility URI must be assembled. The following E-utility URI fetches the first four sequences from the previous query in FASTA format: https://eutils.ncbi.nlm.nih.gov/entrez/eutils/efetch.fcgi? db=nucleotide&id=1509580163, 1509580026, 1509580024, 1509580022&rettype=fasta&retmode=text. ESearch, ELink and EPost queries can be stored on the Entrez History server (Fig. 1B). Such queries return a reference as part of the result, consisting of a WebEnv string and a query_key number. The WebEnv value is not static and stored only temporarily, while the query_key increments for each query using the same WebEnv. Together, these values can be used in subsequent queries to reference a prior query. Using the History servers can reduce the amount of data to download. For example, queries fetching large datasets can store the preceding search query and thereby prevent the downloading of large numbers of UIDs. Another use is to combine queries using E-Utilities on the NCBI severs, such as via Elink queries. NCBI enforces a limit of three requests per second to E-Utilities. With an NCBI API key, this limit can be raised to ten requests per second (https://www.ncbi.nlm.nih.gov/books/NBK25500/). By default, Entrezpy enforces the lower limit, but if an NCBI API key is used or stored as environmental variable (see Supplementary Material for details), the upper limit is used.

Entrezpy supports the E-Utilities EFetch, ESearch, ELink, ESummary and EPost. The E-Utilities ESpell (spelling suggestions), EInfo (database statistics), ECitMatch (batch citation searching in PubMed) and EGQuery (global ESearch) are currently not supported since they can be either assembled using existing functions or have a very broad usage. Entrezpy is not primarily intended to replace an NCBI website search, but to run queries for a specific problem. The Entrezpy functions implemented use the same parameters as those described in the Entrez manual. NCBI limits query and retrieval sizes. For example, downloading summaries in JSON format is limited to 500 summaries at a time. In such cases, queries must be split into several requests to obtain the whole requested dataset (Fig. 1C). Entrezpy automates these steps, enabling the easy assembly of complex E-Utility queries to search the Entrez databases and download datasets. Entrez History server responses can be used to link queries, analogous to piping commands on UNIX systems (Fig. 1D). Entrezpy is designed to analyze the response from each request as soon as it is received, allowing the implementation of checkpoints when handling large datasets, for example, whether to resume after aborts or errors. For the Efetch, Esummary and ESearch functions we added the parameter req_size that sets the size of requests within a query. We observed that in some cases connection timeout errors can be solved by setting a smaller request size for the query. Entrezpy uses no threading by default to download datasets but can use multithreading.

The class Conduit simplifies the assembly of complex queries (Fig. 1). Internally, Entrezpy assigns each query and requests a unique identifier. This allows Entrezpy to cache queries and results, thereby enabling to access data from an earlier query as parameters for a new query. We implemented this caching approach in Conduit, in which all Conduit instances share the same cache and are cleared if the pipeline is finished or aborted. In addition, Entrezpy result classes can assemble and return parameters that can be used as input parameters for other Entrezpy functions, such as an Esearch result return input parameters for Efetch. Together with the ability to cache results, this allows Entrezpy to create complex queries. In Conduit, such a series of queries is called a pipeline (Fig. 1E). Queries can be added to a Conduit pipeline either as parameter or as dependency (Fig. 1E). A dependency is a query ID from an earlier query and Conduit will obtain the corresponding parameters from the cache. If query parameters and a dependency are been passed to a Conduit query, the parameters overwrite the corresponding parameter obtained via the dependency.

Entrezpy checks for errors in parameters, during requests and after receiving the response from NCBI. If erroneous parameter combinations or values are recognized, Entrezpy aborts. During a request, Entrezpy checks for connection errors and aborts immediately if the HTTP error 400 is returned (Bad request). For other connection errors, Entrezpy retries the request ten times with a randomized waiting time. For timeout errors, Entrezpy increases its request time in ten steps until the maximum request time of one minute is exhausted. If an error persists after ten retries the query is aborted. All Entrezpy aborts return a log message describing the problem. After receiving the response, Entrezpy checks for error messages in the NCBI response, for example, Entrez database errors. These errors trigger a log message, but the request is technically considered a success and Entrezpy does not abort.

The versatility of Entrezpy is based on the use of virtual functions and modular design. We implemented a default analyzer for all E-Utilities. However, the default Efetch analyzer is very basic and prints results to the standard output. This is deliberate, since an analyzer for an Efetch request is usually the last step in query. Given the numerous possibilities, databases and formats available, finalizing and adjusting an appropriate Efetch analyzer is best left to the pipeline developer. Creating a specific analyzer requires the implementation of only two virtual functions of the Entrezpy analyzer base class, specifically the methods to handle errors and the result. Therefore, a new and highly specific analyzer for a specific dataset can be written without the need to adjust the whole request process.

Entrezpy has been designed ‘to do one thing and do it well’. It enables the querying and downloading data from the Entrez databases, one of the largest life sciences data repositories, while giving a developer the freedom to easily integrate specific analysis functions.

## Funding

This work was supported by an ARC Australian Laureate Fellowship [FL170100022 to E.C.H.].

*Conflict of Interest*: none declared.

## References

[btz385-B1] Cock P.J.A. et al (2009) Biopython: freely available Python tools for computational molecular biology and bioinformatics. Bioinformatics, 25, 1422–1423. 19304878 10.1093/bioinformatics/btp163 PMC2682512

[btz385-B2] Huerta-Cepas J. et al (2016) ETE 3: reconstruction, analysis, and visualization of phylogenomic data. Mol. Biol. Evol., 33, 1635–1638. 26921390 10.1093/molbev/msw046 PMC4868116

[btz385-B3] Kans J. (2016) *Entrez Direct: E-utilities on the UNIX Command Line*. National Center for Biotechnology Information, Bethesda, MD, USA.

[btz385-B4] NCBI Resource Coordinators (2016) Database resources of the National Center for Biotechnology Information. Nucleic Acids Res., 45, D12–D17. 27899561 10.1093/nar/gkw1071 PMC5210554

